# Roles of lncRNAs in NF-κB-Mediated Macrophage Inflammation and Their Implications in the Pathogenesis of Human Diseases

**DOI:** 10.3390/ijms25052670

**Published:** 2024-02-25

**Authors:** Jae-Joon Shin, Jeongkwang Park, Hyeung-Seob Shin, Imene Arab, Kyoungho Suk, Won-Ha Lee

**Affiliations:** 1 School of Life Sciences, BK21 Plus KNU Creative BioResearch Group, Kyungpook National University, Daegu 41566, Republic of Korea; wowns1124@knu.ac.kr (J.-J.S.); wjdrhkd28787@gmail.com (J.P.); gudals3963@naver.com (H.-S.S.); arabimene07@gmail.com (I.A.); 2 Department of Pharmacology, Brain Science and Engineering Institute, BK21 Plus KNU Biomedical Convergence Program, School of Medicine, Kyungpook National University, Daegu 41944, Republic of Korea; ksuk@knu.ac.kr

**Keywords:** human diseases, inflammation, lncRNA, macrophage, NF-κB

## Abstract

Over the past century, molecular biology’s focus has transitioned from proteins to DNA, and now to RNA. Once considered merely a genetic information carrier, RNA is now recognized as both a vital element in early cellular life and a regulator in complex organisms. Long noncoding RNAs (lncRNAs), which are over 200 bases long but do not code for proteins, play roles in gene expression regulation and signal transduction by inducing epigenetic changes or interacting with various proteins and RNAs. These interactions exhibit a range of functions in various cell types, including macrophages. Notably, some macrophage lncRNAs influence the activation of NF-κB, a crucial transcription factor governing immune and inflammatory responses. Macrophage NF-κB is instrumental in the progression of various pathological conditions including sepsis, atherosclerosis, cancer, autoimmune disorders, and hypersensitivity. It orchestrates gene expression related to immune responses, inflammation, cell survival, and proliferation. Consequently, its malfunction is a key contributor to the onset and development of these diseases. This review aims to summarize the function of lncRNAs in regulating NF-κB activity in macrophage activation and inflammation, with a particular emphasis on their relevance to human diseases and their potential as therapeutic targets. The insights gained from studies on macrophage lncRNAs, as discussed in this review, could provide valuable knowledge for the development of treatments for various pathological conditions involving macrophages.

## 1. Introduction

Inflammation, a natural and complex biological response, is crucial in defending against infection [1,2]. Yet, chronic or uncontrolled inflammation can be harmful, contributing to the development of various diseases [3]. Diet, exercise, smoking, and stress, as lifestyle factors, can impact bodily inflammation levels, underscoring a healthy lifestyle’s importance in preventing and managing diseases [4]. In some instances, medical treatments target inflammation to ease symptoms and control disease progression [5,6].

Macrophages, innate immune cells with antigen-presenting capabilities, are central to inflammatory responses, significantly impacting acute and chronic inflammation [7]. Macrophages exhibit a dual role in inflammation; pro-inflammatory M1 macrophages drive inflammation, while anti-inflammatory M2 macrophages aid in tissue repair and inflammation resolution [8]. The balance between M1 and M2 macrophages is crucial, as their imbalance can result in immune-related diseases, including chronic inflammation, cancer, hypersensitivity, and autoimmune disorders [9]. This dichotomy is not strictly binary but represents a spectrum of activation states that macrophages can adopt in response to various signals within their microenvironment.

M1 macrophages, induced by IFN-γ alone or in combination with microbial products like lipopolysaccharide (LPS), are potent effectors of the pro-inflammatory response [10]. They produce high levels of pro-inflammatory cytokines (e.g., TNF-α, IL-1β, IL-6), promote Th1 responses, and are highly microbicidal. Their role is crucial in defending against intracellular bacteria and viruses, as well as in tumor suppression. However, the chronic activation of M1 macrophages is implicated in the pathogenesis of autoimmune and inflammatory diseases due to their tissue-damaging effects [11,12]. M2 macrophages are induced by IL-4 and IL-13 and are associated with tissue repair, resolution of inflammation, and immune regulation [5,10]. They produce anti-inflammatory cytokines (e.g., IL-10, TGF-β) and growth factors that promote tissue remodeling, wound healing, and angiogenesis. While essential for recovery from injury, excessive or inappropriate M2 activity can contribute to fibrosis, allergic responses, and the suppression of effective immune responses against tumors [11,12].

An imbalance between M1 and M2 macrophages constitutes a pivotal pathogenetic mechanism in numerous diseases. A predominance of M1 macrophages can lead to chronic inflammatory states, including rheumatoid arthritis, inflammatory bowel disease, and atherosclerosis. In these conditions, sustained inflammation results in tissue damage and drives disease progression [9,13]. Conversely, an excess of M2 macrophages can promote tumor growth and metastasis and contribute to fibrotic diseases and certain infections by failing to eliminate pathogens adequately [8,14].

Given their central role in both the promotion and resolution of inflammation, as well as in tissue repair, macrophages present a valuable target for therapeutic intervention. Strategies to modulate macrophage activity and polarization could offer new avenues for treating a wide range of diseases. For instance, therapies aimed at promoting M1 activity could enhance anti-tumor immunity or combat chronic infections, while strategies to boost M2 functions might aid in tissue repair and resolution of inflammation [8,15]. Rebalancing the M1/M2 ratio could provide therapeutic benefits in diseases characterized by an imbalance in macrophage polarization. Therefore, targeting macrophage activity represents a promising approach for the development of novel therapeutic strategies aimed at a variety of inflammatory and immune-related diseases.

The nuclear factor kappa-light-chain-enhancer of activated B cells (NF-κB) is pivotal in regulating inflammation, particularly in macrophages. NF-κB, comprising p50, p52, p65 (RelA), RelB, and c-Rel subunits, activates genes related to immune response, inflammation, and cell survival by forming homodimers and heterodimers [16]. Activation of NF-κB involves the nuclear translocation of the p65/p50 heterodimer, whereas the p50 homodimer acts as an inhibitor [17]. NF-κB activation occurs via two primary pathways: the canonical (classical) and the non-canonical (alternative) [18]. Differing in activation mechanisms and stimuli, these pathways enable precise and dynamic NF-κB regulation. While the canonical pathway primarily mediates rapid and transient NF-κB activation in response to pro-inflammatory stimuli, the non-canonical pathway is involved in the regulation of adaptive immune responses, lymphoid organ development, and maintenance of immune homeostasis. Induced by cytokines like tumor necrosis factor (TNF)-α and interleukin (IL)-1, the canonical pathway involves TNF receptor-associated factor 6 (TRAF6) and the IKK complex (IKKα, IKKβ, IKKγ), leading to IκB phosphorylation. Subsequent degradation of IκB liberates p65/p50 heterodimer for nuclear translocation. Initiated by TNF receptor superfamily members like lymphotoxin β receptor (LTβR) or B cell-activating factor receptor (BAFF-R), the non-canonical pathway is then mediated by NF-κB-inducing kinase (NIK) and IKKα for phosphorylation and processing of p100 to generate p52. The resulting RelB/p52 heterodimers then translocate to the nucleus for transcriptional activation [18]. Dysregulation in either pathway can lead to numerous inflammatory and immune-related diseases.

**Figure 1:**
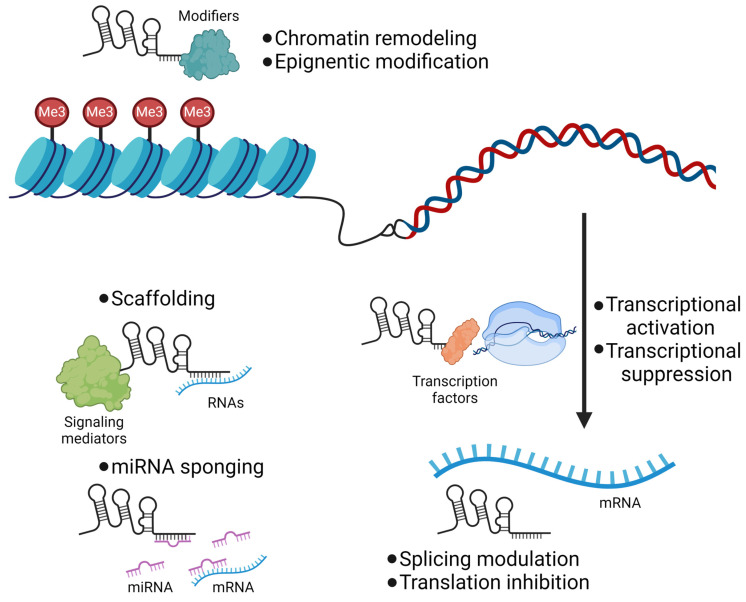
**General action mechanisms of lncRNA.** LncRNAs interact with chromatin-remodeling complexes or histone-modifying enzymes to facilitate epigenetic modifications. Their interaction with transcription factors regulates the transcriptional activity of target genes. Interactions with mRNA affect post-transcriptional regulations, including splicing and translation. LncRNAs can also serve as scaffolds for interactions among signaling mediators and various RNAs, including small RNAs, miRNAs and mRNAs. Finally, the miRNA sponging action of lncRNAs may regulate the stability of target mRNAs.

Long noncoding RNAs (lncRNAs) constitute a heterogeneous group of RNA molecules exceeding 200 nucleotides in length that do not encode proteins, yet are pivotal in regulating a myriad of biological functions [19]. These molecules are characterized by their complex roles in chromatin organization, gene transcription, RNA splicing, and epigenetic modifications [20,21,22]. LncRNAs influence chromatin accessibility and gene transcriptional activity by directing chromatin-modifying enzymes to specific genomic regions (Figure 1). This action can either enhance or suppress gene expression, contingent upon the context and the nature of the modifications implemented [20,23]. The synthesis of lncRNAs mirrors that of mRNAs, encompassing transcription primarily by RNA polymerase II, capping, splicing, and polyadenylation [24]. Nonetheless, lncRNAs generally exhibit reduced splicing efficiency and a diminished rate of nuclear-to-cytoplasmic export relative to mRNAs. Their genomic distribution is varied, including intergenic regions, areas overlapping with, or antisense to, protein-coding genes [19]. This diversity implies a broad spectrum of mechanisms through which lncRNAs can modulate gene expression and cellular functionality.

In their mechanisms of action, lncRNAs engage in various cellular processes. They function as guides, recruiting chromatin-modifying enzymes to specific genomic locations, thereby reshaping the epigenetic landscape and directly influencing gene expression at the DNA level [23,25]. As decoys, lncRNAs can attract and sequester transcription factors or other proteins away from their DNA targets, indirectly impacting transcription [21,22,26]. Moreover, serving as scaffolds, lncRNAs facilitate the assembly of multiple proteins into ribonucleoprotein complexes, enabling the orchestration of intricate molecular operations. Additionally, lncRNAs partake in post-transcriptional regulation by interacting with signaling mediators and by absorbing microRNAs (miRNAs) [27]. Through miRNA sequestration, lncRNAs obstruct miRNA attachment to target mRNAs, thereby moderating post-transcriptional gene silencing and influencing mRNA stability and translation.

**Figure 2:**
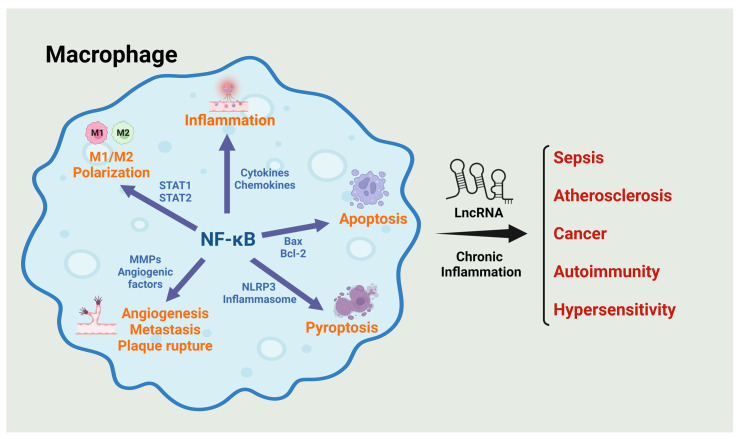
**Role of lncRNAs and macrophage NF-κB in the pathogenesis of human diseases.** In macrophages, lncRNAs regulate NF-κB activity either directly or indirectly, influencing various processes that lead to inflammation, apoptosis, pyroptosis, and M1/M2 polarization. Additionally, the activation of NF-κB in macrophages affects neighboring cells and the extracellular matrixes, regulating angiogenesis and metastasis in cancer development, as well as plaque rupture in atherosclerosis. These macrophage-associated events are closely linked to chronic inflammation, which ultimately contributes to the progression of various diseases, including sepsis, atherosclerosis, cancer, autoimmunity, and hypersensitivity.

With the advancement of high-throughput sequencing and bioinformatics tools, a growing number of lncRNAs have been identified as key regulators in macrophage activity, particularly in the regulation of NF-κB signaling pathways. LncRNAs can modulate NF-κB activity through direct interaction with NF-κB itself or its signaling components, or by influencing the expression of genes that are part of the NF-κB signaling pathway. This regulation can have profound effects on the inflammatory response of macrophages, impacting the development and progression of a wide array of human diseases (Figure 2). This review aims to comprehensively explore the multifaceted roles of lncRNAs in modulating NF-κB activity within macrophages. By highlighting specific examples of lncRNAs that have been implicated in this process, it underscores their potential as biomarkers for disease diagnosis and prognosis, as well as targets for therapeutic intervention.

## 2. LncRNAs That Modulate Macrophage NF-κB Activity in Sepsis

Sepsis is a life-threatening condition marked by a dysregulated immune response to infection, leading to widespread inflammation, organ dysfunction, and potentially organ failure and death [28]. Often caused by bacteria, viruses, fungi, or other pathogens, a prime stimulant is LPS, an endotoxin found in the cell wall of Gram-negative bacteria [29]. The systemic inflammatory response, particularly post-bloodstream infection, triggers an excessive release of pro-inflammatory cytokines and chemokines. These mediators damage the endothelial cells lining the walls of blood vessels, resulting in increased permeability. This, in turn, leads to fluid leakage, tissue swelling, and subsequently edema [30]. Persistent inflammation can impact multiple organs and systems, with cytokines like TNF-α being key mediators of sepsis [31]. Commonly affected organs include the lungs, heart, kidneys, liver, and even the central nervous system.

NF-κB-mediated inflammation in macrophages, followed by M1 polarization, is pivotal in the pathogenesis of sepsis [32]. Pattern recognition receptors, including Toll-like receptors (TLRs), allow macrophages to identify pathogen-associated molecular patterns on invading microorganisms [29]. Activation of NF-κB, triggered by LPS interaction with TLR4 via the canonical pathway, upregulates numerous pro-inflammatory genes, such as cytokines, chemokines, adhesion molecules, and enzymes like inducible nitric oxide synthase [33]. NF-κB serves as a primary target for immunomodulatory therapies aimed at regulating inflammation and reestablishing immune equilibrium in many diseases, including sepsis. Such strategies might involve inhibiting NF-κB activation or employing targeted therapies to neutralize specific pro-inflammatory cytokines.

**Figure 3:**
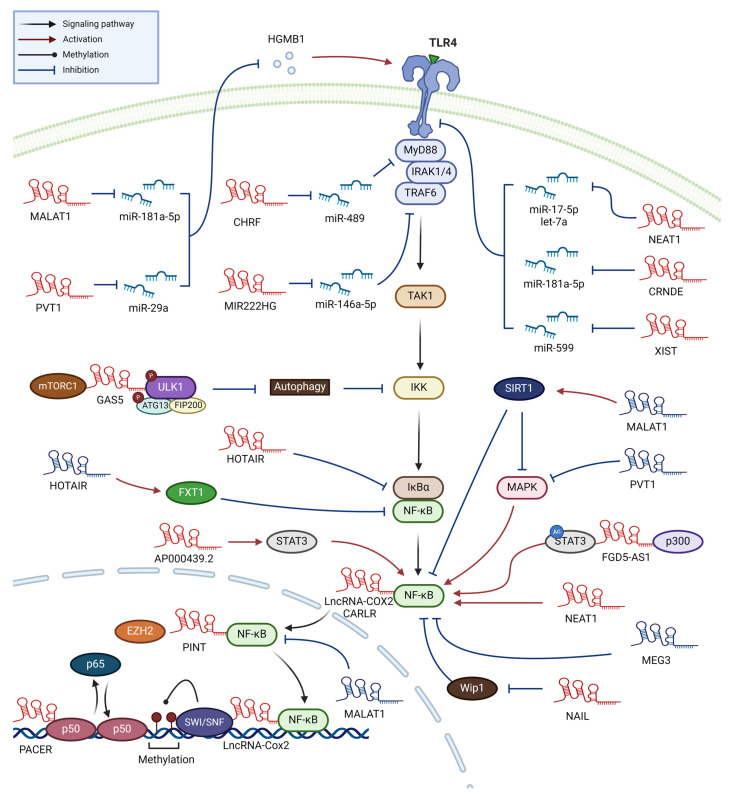
**Mechanisms triggered by macrophage lncRNAs in the regulation of NF-κB activity.** Various lncRNAs that regulate the NF-κB activation pathway are depicted. Activating effects are represented by red arrows, while inhibiting effects are shown with blue arrows. LncRNAs that enhance NF-κB activity are indicated in red, while those that suppress NF-κB activity are shown in blue.

The lncRNA nuclear paraspeckle assembly transcript 1 (NEAT1), confined to the nucleus, is instrumental in forming paraspeckles, subnuclear structures involved in antiviral responses [34]. The levels of NEAT1 are increased by more than two-fold in the sera of sepsis patients [35,36,37,38]. Induced by LPS in the human monocytic leukemia cell line THP-1, NEAT1 enhances inflammatory responses by sponging miR-17-5p, thereby stabilizing TLR4 mRNA (the miR-17-5p/TLR4 axis) (Figure 3) [38]. In Kupffer cells and the murine macrophages, LPS-induced NEAT1 promotes inflammatory activities via the Let-7q/TLR4 axis [35,36,37,39]. Additionally, NEAT1 also facilitates M1 polarization in macrophages through the miR-125a-5p/TRAF6/TGF-β-activated kinase 1 (TAK1) axis [40], underscoring its potential as a therapeutic target for sepsis.

Metastasis-associated lung adenocarcinoma transcript 1 (MALAT1), a multifunctional lncRNAs in macrophages, is extensively studied. In late-onset sepsis patients, increased blood MALAT1 levels correlate with disease severity [41]. The expression of MALAT1 increases in activated macrophages, exhibiting an elevation greater than two-fold in mouse peripheral blood mononuclear cells (PBMCs) and more than six-fold in THP-1-derived macrophages, especially following LPS treatment [41,42]. Animal studies show that MALAT1 expression surges with sepsis induction; reducing MALAT1 lessens inflammation and mortality [41,43,44,45], possibly by suppressing M1 and enhancing M2 macrophage polarization [45]. In an LPS-induced septic lung injury model, intravenous MALAT1-specific small interfering RNA (siRNA) decreases inflammatory cytokines and immune cells in bronchoalveolar lavage fluid by inhibiting the p38 mitogen-activated protein kinase (MAPK)/p65 pathway [44].

However, several reports have shown contradictory evidence, indicating a significant decrease in MALAT1 expression accompanied by an increase in hsa-miR-346 levels in patients with sepsis. Activated human and mouse macrophages downregulate MALAT1 expression in an NF-κB-dependent manner [42,46]. MALAT1 interacts with NF-κB, inhibiting its DNA-binding activity and, consequently, the expression of pro-inflammatory cytokines [42]. Furthermore, MALAT1 modulates macrophage proliferation by sequestering hsa-miR-346, thereby stabilizing the mRNA of small mothers against decapentaplegic homolog 3 (SMAD3). SMAD3 is a receptor-regulated signaling adaptor that is activated by serine kinases. These conflicting findings underscore the need for future research on MALAT1’s role in sepsis.

In sepsis patients, the levels of long noncoding RNA plasmacytoma variant translocation 1 (PVT1) are elevated, showing an increase of more than two-fold compared to the healthy control group. This elevation correlates with increased pro-inflammatory mediators and survival rates. [47,48]. LPS induces PVT1 expression in THP-1 cells, which in turn amplifies NF-κB activity via p38 stimulation [49]. Elevated PVT1 expression, promoting M1 polarization through the miR-29a/high-mobility group box 1 (HMGB1) axis, is observed in heart-infiltrating macrophages of septic mice [50]. HMGB1, released from the cells, can activate TLR4 in both autocrine and paracrine manners [51]. PVT1 is also highly expressed in osteoarthritis patients’ serum and in the LPS-stimulated C28/12 chondrocyte cell line, activating the TLR4/NF-κB pathway via the miR-93-5p/HMGB1 axis [52]. Additionally, PVT1 levels rise in myocardial tissues and heart-infiltrating macrophages during sepsis-induced myocardial injury [50].

The expression level of lncRNA MEG3 is significantly reduced in patients with sepsis, and this reduction has prognostic significance [53]. In macrophages, MEG3 overexpression inhibits LPS-induced apoptosis by downregulating BAX and upregulating Bcl-2. It also suppresses inflammatory factor expression by inhibiting NF-κB signaling [53]. This suggests that the reduced MEG3 expression may exacerbate sepsis by increasing inflammation and inhibiting apoptosis in macrophages. Further research is needed to elucidate MEG3’s role in sepsis.

In sepsis patients, the lncRNA colorectal neoplasia differentially expressed (CRNDE) exhibits elevated expression in peripheral blood, with higher levels correlating to improved survival rates [54]. CRNDE intensifies LPS-induced NF-κB activation and subsequent pro-inflammatory cytokine release in THP-1 cells via the miR-181-5p/TLR4 axis [54].

These reports highlight the intricate relationship between lncRNAs and NF-κB in the context of sepsis, impacting inflammatory activation and macrophage polarization. The influence of these lncRNAs on cytokine release, cell polarization, and apoptosis is notable, and their varied expression in sepsis patients suggests potential as biomarkers. Targeting these lncRNAs to regulate NF-κB activation offers promising avenues for immunomodulatory therapies to manage inflammation and restore immune balance in sepsis. However, the contrasting roles of specific lncRNAs, like MALAT1, necessitate further research. A deeper understanding of these lncRNAs’ roles could lead to innovative diagnostic and therapeutic strategies, improving management and outcomes in sepsis.

## 3. LncRNAs That Modulate Macrophage NF-κB Activity in Atherosclerosis

Macrophages are central in atherosclerosis development, marked by arterial plaque build-up. The process initiates with low-density lipoprotein (LDL) cholesterol accumulation in arterial walls, undergoing oxidation and eliciting inflammation [55,56]. Modified LDL attracts monocytes from blood, transforming into macrophages in the arterial wall. These macrophages consume oxidized LDL (oxLDL), forming lipid-laden foam cells and creating fatty streaks, early atherosclerosis signs [57,58]. M1 macrophages exacerbate inflammation by releasing cytokines, attracting more immune cells [59,60]. Chronic inflammation leads to fibrous cap formation over plaques and extracellular matrix accumulation. Macrophages also degrade this matrix, heightening plaque instability and increasing heart attack and stroke risks [57,61]. Macrophages can also contribute to the resolution of inflammation and healing processes [62]. In atherosclerosis, inflammation resolution is overshadowed by ongoing inflammation and plaque growth.

NF-κB, activated by stimuli such as oxidative stress, cytokines, and oxLDL, exacerbate atherosclerosis by promoting lipoprotein uptake, foam cell formation, and attracting more immune cells [57,63]. This activation also destabilizes plaques by encouraging matrix metalloproteinase (MMP) secretion, increasing plaque rupture risks [64]. Chronic NF-κB activation sustains the inflammation characteristics of advanced atherosclerosis in conditions like coronary artery diseases (CADs) and myocardial infarction (MI) [65]. Given its crucial role in macrophage activation atherosclerosis progression, NF-κB presents a potential target for therapies aimed at reducing inflammation and slowing atherosclerosis progression [66].

Increased NEAT1 expression levels in the PBMCs and sera of atherosclerosis patients have been noted [67,68]. The expression level of NEAT1 was found to be increased by more than two-fold in PBMCs of CAD patients [67]. NEAT1, induced by oxLDL in THP-1 cells, contributes to pro-inflammatory responses by enhancing p65 phosphorylation, followed by paraspeckle formation [69,70]. It is also induced in bone marrow-derived macrophages (BMDMs) treated with titanium particles and promotes NF-κB activation, NLRP3 inflammasome formation, and M1 polarization via the miR-188-5p/Bruton’s tyrosine kinase (BTK) axis [71]. NEAT1 also stimulates pro-inflammatory cytokine and reactive oxygen species (ROS) production and subsequent foam cell formation by sponging miR-342-3p in THP-1 cells [70] or miR-128 in the murine macrophage-like cell line RAW264.7 [72]. These reports agree with NEAT1 being expressed in activated macrophages and enhancing pro-inflammatory changes. One contradicting study, however, reported decreased NEAT1 in the PBMCs of post-MI patients and enhanced macrophage inflammation in NEAT1-knockout mice (Table 1) [73].

Elevated lncRNA PVT1 levels have been detected in the serum of atherosclerosis patients [74]. Inhibiting PVT1 in animal models reduces atherosclerotic plaques by increasing HDL levels and suppressing the MAPK/NF-κB pathway and pro-atherogenic factors [74]. In serum samples of atherosclerosis patients and during oxLDL-induced THP-1 cell foam cell differentiation, there is a notable increase in lncRNA small nucleolar RNA host gene (SNHG)16 and a decrease in miR-17-5p [75]. SNHG16 amplifies macrophage proliferation and pro-inflammatory responses in atherosclerosis through the miR-17-5p/NF-κB axis [75].

LncRNA X-inactive specific transcript (XIST), known for its role in X-chromosome inactivation, has been found to be elevated more than two-fold in the serum of atherosclerosis patients, oxLDL-treated vascular smooth muscle cells, and the U937 human monocytic leukemia cell line [76]. XIST influences atherosclerosis by promoting proliferation and inhibiting apoptotic cell death through the miR-599/TLR4 axis [76]. This finding aligns with other studies that show that apoptosis inhibition aggravates atherogenesis by increasing macrophage proliferation and plaque formation [77,78].

LncRNA H19 is found at elevated levels in the serum of atherosclerosis patients [79,80,81,82]. OxLDL stimulates H19 expression in macrophages [83], aorta vascular smooth muscle cells [79,80], and human umbilical vein endothelial cells (HUVECs) [84]. In macrophages, H19 augments oxLDL-induced lipid accumulation, ROS generation, and NF-κB activation [83,85]. Similarly, in HUVECs, H19 heightens NF-κB activation by increasing p38 and p65 activity [86]. These findings suggest that H19 could be a promising therapeutic target for atherosclerosis treatment.

In atherosclerosis and CAD patients, MALAT1 levels rise more than two-fold and subsequently fall after treatment [87,88,89]. MALAT1 impacts various macrophage processes like foam cell formation, autophagy, and pyroptosis [90,91,92]. OxLDL prompts NF-κB-dependent MALAT1 expression in THP-1 cells. MALAT1 then enhances lipid uptake and foam cell formation by promoting scavenger receptor CD36 expression [88,90,91]. MALAT1 also enhances NF-κB activation and subsequent inflammation by sponging miR-330-5p [91]. Further, oxLDL-induced autophagy in macrophage is mediated by MALAT1, which activates the MAPK/NF-κB pathway and inhibits sirtuin 1 (SIRT1), a key transcription factor deacetylase [92,93]. NLRP3 inflammasome-mediated pyroptosis, a programmed cell death as a defense mechanism against intracellular pathogens, is also influenced by MALAT1 [94]. In diabetic atherosclerosis models, a cinnamic acid derivative reduces inflammasome activation and pyroptosis by suppressing MALAT1 [95]. Extracellular vesicles (EVs) such as exosomes are crucial for cell-to-cell communication, transferring proteins and lncRNAs [96]. M1 macrophages have been found to release MALAT1-containing EVs, which regulate myocyte proliferation and angiogenesis in MI models [97]. These findings underscore MALAT1’s role in atherosclerosis: it is upregulated in activated macrophages and influences various processes including lipid uptake, foam cell formation, and cell death.

However, contrary reports exist regarding the role of MALAT1 in atherosclerosis. It was observed that in atherosclerosis patients and oxLDL-treated THP-1 cells, MALAT1 levels decrease [88]. Reduced MALAT1 leads to increased lipid and total cholesterol accumulation in THP-1 cells via the miR-17-5p/ATP-binding cassette subfamily A member 1 (ABCA1) axis [88]. ABCA1 is known to facilitate cholesterol efflux, thereby reducing foam cell formation [98]. Additionally, MALAT1 deficiency in certain mouse models has been linked to accelerated macrophage inflammation and atherosclerosis [99]. Exosomal MALAT1 from oxLDL-treated HUVECs promotes a transition from M1 to M2 macrophages [100]. These findings suggest potential anti-atherogenic properties of MALAT1, highlighting the need for further research to clarify its role in atherosclerosis.

Notably, the expression levels of lncRNA HOX transcript antisense intergenic RNA (HOTAIR) are decreased in the peripheral blood lymphocytes of atherosclerosis patients and oxLDL-treated RAW264.7 cells [101]. HOTAIR overexpression reduces pro-inflammatory cytokine expression while boosting anti-inflammatory cytokines, achieved by inhibiting NF-κB activity. This suppression occurs through HOTAIR’s enhancement of fragile X-related protein 1 (FXR1) levels, a protein moving between the nucleus and cytoplasm and associating with polyribosomes [101,102].

These reports underscore the complex relationship between various lncRNAs and macrophage NF-κB in atherosclerosis. These lncRNAs impact crucial aspects such as lipid uptake, foam cell formation, inflammation, and cell death in macrophages. Given their link to NF-κB activation, targeting these lncRNAs for NF-κB modulation presents a promising approach to managing atherosclerosis by restoring immune equilibrium and curbing inflammatory activation. It is intriguing that certain lncRNAs, such as MALAT1 and HOTAIR, have been identified to play conflicting roles in atherosclerosis. Variations in the stages of atherosclerosis or CAD examined, the measurement techniques utilized, environmental factors, or the experimental model systems employed could account for these discrepancies. Alternatively, the overall impact of these lncRNAs may differ based on the dominant signaling pathways activated in particular contexts or disease states. Despite the conflict, their significant influence on macrophage function and disease progression is evident. Further research is essential to unravel the full potential of these lncRNAs in atherosclerosis treatment.

**Table 1:** **LncRNAs regulating macrophage NF-κB activity in diseases.** LncRNAs that enhance NF-κB activity are indicated in red, while those that suppress NF-κB activity are shown in blue.

LncRNA	Disease	Target	Function	Refs.
Group 1. LncRNAs directly affecting NF-κB activity				
**CARLR**	Celiac disease	p65	LPS-induced, regulates p65 translocation, promotes pro-inflammatory cytokine production	[103]
**COX2**	Neuroinflammation	p65	LPS-induced, promotes p65 translocation, enhances inflammasome formation, suppresses autophagy	[104]
**COX2**	SLE	SWI/SNF/NF-κB	LPS-induced, enhances the expression of NF-κB-induced late inflammatory genes	[105]
**MALAT1**	Sepsis	p65/p50	LPS-induced, retrains NF-κB from promoter, regulates pro-inflammatory cytokine expression	[42]
**PACER**	Cancer	p50	LPS-induced, sequesters p50 and promotes p300-mediated histone acetylation, enhances COX2 expression	[106]
**PINT**	Cancer	p65 and EZH2	LPS-induced, bridges p65 and EZH2, activates TNFα transcription	[107]
Group 2. LncRNAs affecting pathways that regulate NF-κB activity				
**CHRF**	Cancer	miR-489/MyD88	Silica-induced, promotes inflammatory responses and fibrosis	[108]
**CRNDE**	Cancer	miR-181a-5p/TLR4	LPS-induced, promotes inflammatory responses. Also increased in AML and IgA nephropathy	[54,109]
**MALAT1**	Atherosclerosis	SIRT1/MAPK	ox-LDL-induced, promotes autophagy, reduces apoptosis	[93]
**MIR222HG**	Allergic rhinitis	miR-146a-5p/TRAF6	Deceased in patients, causing the dominance of type 2 response. Promotes M1 and suppresses M2 polarization	[110]
**NAIL**	Ulcerative colitis	Wip1	LPS-induced, promotes p65 phosphorylation by blocking Wip1 action, increases inflammatory response	[111]
**NEAT1**	Osteolysis	miR-188-5p/BTK KLF/BTK	Induced by titanium particles, activates inflammasome and NF-κB, enhances M1 polarization	[71]
**NEAT1**	Sepsis	miR-17-5p/TLR4	LPS-induced, promotes inflammatory response by stabilizing TLR4 mRNA	[38]
**NEAT1**	Sepsis	let-7a/TLR4	LPS-induced, promotes inflammatory response by stabilizing TLR4 mRNA	[35]
**NKILA**	Asthma	IκB	Anti-inflammation by inhibiting IκB phosphorylation, induces M2 polarization	[112,113]
**PVT1**	Sepsis	miR-29a/HMGB1/TLR4	LPS-induced, promotes inflammation and M1 polarization. Also increased in osteoarthritis patients	[50,52]
**SNHG1**	Cancer/Sepsis	HMGB1/TLR4	Enhances TLR4 signaling by interaction with HMGB1, promotes M1 polarization	[114]
**XIST**	Atherosclerosis	miR-599/TLR4	OxLDL-induced in macrophages/vascular smooth muscle cells, promotes proliferation and suppresses apoptosis	[76]
Group 3. LncRNAs affecting NF-κB activity with unknown mechanism of action				
**DCST1-AS1**	Cancer	p65	Activates NF-κB signaling pathway in both cancer cells and macrophages. Promotes M2 polarization	[115]
**FTX**	Cirrhosis	NF-κB	Anti-inflammatory function. Decreased in patients, causing enhancement in inflammation	[116]
**HOTAIR**	Cancer	IκBα	LPS-induced, activates NF-κB by degrading IκB, regulates metabolic reprogramming by inducing GLUT1	[117]
**HOTAIR**	Atherosclerosis	FXR1	Anti-inflammation by inactivating NF-κB via FXR1. Decreased in patients and oxLDL-treated macrophages.	[101,102]
**NEAT1**	Atherosclerosis	p65, ERK	ox-LDL-induced, regulates p65 and ERK phosphorylation, promotes TNF-α secretion.	[34,69]
**MEG3**	Sepsis	p65	Decreased in sepsis patients. LPS-induced, inhibits p65 phosphorylation. Downregulates inflammation and apoptosis.	[53]
**SNHG16**	Atherosclerosis	miR-17-5p	ox-LDL-induced, promotes proliferation and inflammatory response. Also increased in cancer and diabetes	[75,118,119]
Group 4. LncRNAs transferred to macrophages via exosomes or EVs				
**FGD5-AS1**	Cancer	P300/STAT3/NF-κB	Contained in exosomes from pancreatic cancer cells, activates STAT3/NF-κB, promotes M2 polarization	[120]
**AP000439.2**	Cancer	STAT3, P65	Contained in exosomes from cancer cells, promotes macrophage M2 polarization	[121]
**GAS5**	Allergic rhinitis	mTORC1/ULK1/ATG13	Activates NF-κB and promotes M1 polarization by suppressing autophagy-dependent degradation of IKKa/b	[122]
**HOTTIP**	Cancer	-	Contained in M1-derived exosomes. Suppresses cancer growth via TLR5 activation. Promotes M1 polarization	[123]
**MALAT1**	Acute pancreatitis	miR-181a-5p /HNGB1/TLR4	Carried by EVs originating from pancreatic cancer cells, promotes M1 polarization	[124]

## 4. LncRNAs That Modulate Macrophage NF-κB Activity in Cancer

The role of macrophage inflammation in cancer is multifaceted and contradictory. M1 macrophages, typically anti-tumorigenic, can attack tumor cells and stimulate immune responses. Conversely, M2 macrophages often aid tumor growth by supporting angiogenesis, suppressing immune responses, and facilitating tissue remodeling [125]. Generally, tumor-associated macrophages (TAMs) exhibit an M2 phenotype, supporting tumor growth and metastasis and contributing to an immunosuppressive tumor environment [126,127]. Given their significant impact on cancer progression, TAMs are being investigated as therapeutic targets, with strategies focusing on inhibiting their tumor-promoting functions or reprogramming them to combat tumors.

The activation of NF-κB in macrophages plays a crucial role in cancer development and progression. In TAMs, NF-κB activation leads to the production of cytokines, growth factors, and enzymes that promote tumor growth and suppress anti-tumor immune responses [128,129]. NF-κB can also alter the immune microenvironment, potentially inducing immune checkpoint molecules that weaken the immune response against tumors [130]. Additionally, NF-κB-activated macrophages can produce angiogenic factors, aiding tumor vascularization [131,132,133]. They can also stimulate matrix metalloproteinases (MMPs), breaking down extracellular matrix barriers and facilitating cancer cell spread [134,135]. Thus, macrophage NF-κB is implicated in various aspects of cancer progression and targeting macrophage NF-κB has emerged as a prominent focus in cancer treatment strategies [18].

LncRNA DC-STAMP domain containing 1-antisense 1 (DCST1-AS1) has been investigated in various cancers, including gastric, colorectal, cervical, breast, glioblastoma, endometrial, and HCC [136,137,138,139,140,141,142]. In these cancers, increased DCST1-AS1 expression correlates with larger tumors and shorter survival and DCST1-AS1 promotes cancer cell proliferation and metastasis, and inhibits apoptosis, by sponging miRNAs [136,137,138,139,140,141,142]. Notably, in oral squamous cell carcinoma, DCST1-AS1 advances tumor progression by enhancing NF-κB activity in cancer cells and macrophages [115]. The expression of DCST1-AS1 showed a more than three-fold increase in oral squamous cell carcinoma cells compared to normal cells [115]. Elevated DCST1-AS1 in cancer cells and M2 macrophages is linked to tumor growth and cancer cell proliferation. NF-κB antagonists revealed that DCST1-AS1 enhances cancer progression and M2 macrophage polarization through NF-κB-mediated mechanisms [115].

The lncRNA FGD5 antisense RNA 1 (FGD5-AS1) shows elevated levels in non-small-cell lung cancer and pancreatic cancer, correlating with metastasis and poor prognosis [120,143]. FGD5-AS1-containing exosomes from these cancers induce M2 macrophage polarization [120]. FGD5-AS1 links acetyltransferase p300, STAT3, and NF-κB, leading to acetylated STAT3/p65 complex and transcriptional activation [120,144]. STATs are crucial transcription factors in macrophage polarization, with STAT1 being integral to M1, and STAT3/6 to M2 polarization. [145]. In cervical cancer, FGD5-AS1, via the miR-129-5p/bone marrow stromal cell antigen 2 (BST2) axis, promotes tumor growth and M2 polarization [146]. BST2, a lipid raft-associated protein, is implicated in cell proliferation and immune response [147,148]. Collectively, FGD5-AS1 augments tumor growth by enhancing cancer progression and M2 macrophage polarization.

The lncRNA AP000439.2 has recently been identified as a prognostic marker for renal cell carcinoma (RCC) patient survival [149,150]. Exosomes from human RCC cell lines have been shown to induce M2 polarization in co-cultured THP-1 cells [121]. AP000439.2 promotes M2 polarization through the phosphorylation of STAT3 and the NF-κB p65 subunit, which, in turn, enhances the migration potential of cocultured cancer cell lines. The impact of exosomal AP000439.2 on macrophage M2 polarization and RCC growth has been confirmed in a xenograft tumor mouse model [121].

LncRNA Five Prime to Xist (FTX), an evolutionarily conserved regulator of XIST expression, is associated with various conditions including malignancies, endometriosis, and stroke, functioning through miRNA sponging [151,152]. Liu et al. observed decreased FTX levels in cirrhosis patients, linking them to abnormal activation of CD14+ CD16+ monocytes via the miR-545/T cell immunoglobulin and mucin domain 3 (Tim-3) axis [116,153]. Moreover, FTX suppression in THP-1 cells increases NF-κB activity and pro-inflammatory cytokine expression, suggesting that a reduction in FTX might accelerate tumor progression by enhancing inflammation in the tumor microenvironment (TME) [116].

LncRNA HOTAIR, known for its role in gene regulation and epigenetic modifications, is implicated in various human diseases [154]. It is often overexpressed in cancer, contributing to tumor progression, metastasis, and poor prognosis by altering gene expression related to the cell cycle, apoptosis, and metastasis [154,155]. HOTAIR is also associated with central nervous system disorders, fibrosis, and inflammatory conditions, impacting cellular processes and immune responses [156,157,158,159]. It regulates glucose transporter isoform 1 (GLUT1) expression in human neuroblastoma cells and macrophages by stimulating NF-κB activity, suggesting a role in metabolic reprogramming in cancer [117,160]. In addition, inflammatory activation of macrophages triggers HOTAIR expression, which then promotes NF-κB activation and cytokine gene expression by aiding in the degradation of IκBα [117]. HOTAIR’s expression pattern in cancer tissue macrophages remains unexplored and warrants future investigation.

Elevated levels of lncRNA HOXA transcript at the distal tip (HOTTIP) have been observed in AML patients and cell lines, such as U937 and THP-1 [161]. HOTTIP facilitates cell proliferation via the miR-608/DET1- and DDB1-associated 1 (DDA1) axis, with DDA1 being a gene known for its oncogenic properties [161,162]. In squamous cell carcinoma, M1-derived exosomes containing HOTTIP inhibit cancer cell proliferation and induce apoptosis by activating the TLR5/NF-κB pathway [123]. Additionally, exosomal HOTTIP influences the M1 polarization of circulating monocytes [123]. The comprehensive role of HOTTIP in cancer progression remains an area for future exploration.

Cyclooxygenase (COX)2, linked with inflammation in immune cells, is implicated in several cancers [163]. LncRNA p50-associated COX2 extragenic RNA (PACER), located upstream of the COX2 promoter, regulates COX2 expression [106]. PACER, through its association with p50, facilitates p65/p50 heterodimer binding to the COX2 promoter, recruiting p300 histone acetyltransferase [106]. Its expression is upregulated in various cancer tissues, influencing COX2 and PGE_2_ synthesis and cancer cell proliferation, migration, and invasion [164,165,166].

LncRNA cardiac hypertrophy-related factor (CHRF) functions as an oncogene, promoting migration and invasion in various tumor types [167]. In a silica-induced pulmonary fibrosis mouse model, CHRF activates inflammatory and fibrotic pathways via the miR-489/MyD88 and miR-489/SMAD3 axes, with SMAD3 being an adaptor in receptor-regulated signaling [108,168]. CHRF’s pro-inflammatory effects are also observed in LPS-induced acute lung injury [169]. However, its specific role in macrophage inflammation within the TME remains unclear, necessitating further research.

LncRNA SNHG1, commonly overexpressed in various cancers as an oncogene, affects cellular signaling via interactions with miRNAs and signaling regulators [170]. In cholangiocarcinoma cell lines, SNHG1 is associated with increased proliferation and invasion, mediated by NF-κB activation through the miR-140/TLR4 axis, contributing to an inflammatory TME [171]. In an LPS-induced acute lung injury model, SNHG1 is upregulated in M1 polarized THP-1 cells, enhancing NF-κB activation and inflammation through interaction with HMGB1 [114]. However, SNHG1’s specific role in macrophage-related TME remains unexplored.

These findings highlight the intricate relationship between macrophages, lncRNAs, and NF-κB in cancer, affecting cell proliferation, invasion, inflammation, and macrophage polarization. The dichotomy of macrophages, especially TAMs, underscores their potential as therapeutic targets. Their influence extends to inflammation, TME modulation, angiogenesis, and immunosuppression, making them key in the interplay between cancer cells and the immune system. Understanding lncRNA-driven macrophage NF-κB regulation is essential for developing targeted cancer therapies. Despite advances, many aspects of lncRNA functions in cancer and inflammation require further exploration, presenting exciting opportunities for future research and potential therapeutic interventions. 

## 5. LncRNAs That Modulate Macrophage NF-κB Activity in Autoimmunity and Hypersensitivity

NF-κB activation in macrophages plays a pivotal role in the pathogenesis of autoimmunity and hypersensitivity by orchestrating a series of inflammatory responses that can lead to tissue damage and disease progression [172]. In autoimmunity, aberrant NF-κB activation in macrophages contributes to the production of pro-inflammatory cytokines and chemokines, perpetuating a state of chronic inflammation and autoantibody production [173]. This not only disrupts immune tolerance but also promotes the survival and proliferation of autoreactive immune cells. In hypersensitivity reactions, NF-κB activation is crucial for the amplification of immune responses to allergens, leading to exaggerated inflammatory processes [174]. Such activation enhances the expression of molecules involved in antigen presentation and co-stimulation, facilitating the development of Th2 cell responses and the subsequent production of IgE [175]. By driving the inflammatory milieu in both autoimmunity and hypersensitivity, NF-κB activation in macrophages is central to the dysregulation of immune homeostasis and the pathophysiology of these conditions, highlighting its potential as a therapeutic target to modulate immune responses.

A more than two-fold increase in GAS5 levels has been found in exosomes from the nasal mucus of allergic rhinitis patients and a mouse model, promoting macrophage NF-κB activation and M1 polarization [122,176]. GAS5 influences this by inhibiting mTORC1/ULK1/ATG13-mediated autophagy, leading to NF-κB activation [122]. Specifically, GAS5’s activation of NF-κB signaling occurs through the suppression of autophagy-dependent degradation of IKKα/β in macrophages [122]. GAS5 also enhances M1 macrophage polarization, observed in hyperglycemia-induced differentiation in diabetics and in pneumonia-affected children’s macrophages [177,178]. It suppresses the Janus kinase 2/STAT3 pathway, promoting M1 while inhibiting M2 polarization via the miR-455-5p/SOCS3 axis [178]. In diabetes, hyperglycemia-induced GAS5 expression shifts polarization from M2 to M1 by upregulating STAT1 [177]. This shift is significant since M1 macrophage-driven inflammation worsens diabetic wounds, suggesting that reducing GAS5 could facilitate wound healing by promoting an M1-to-M2 transition. On the other hand, microglia exhibit increased GAS5 expression in multiple sclerosis and experimental autoimmune encephalomyelitis (EAE) mouse models [179]. GAS5 hinders M2 polarization by recruiting the polycomb repressive complex 2 (PRC2), suppressing topoisomerase-related function 4 transcription, crucial for M2 polarization [179]. Ito et al. found that reducing GAS5 expression leads to M2b polarization, while increasing it has the opposite effect [180]. GAS5 also plays a role in regulating NLRP3 inflammasome formation in cardiomyocytes [181]. In contrast, GAS5 expression levels are decreased in various cancer tissues [182,183]. This decrease is attributed to GAS5’s ability to induce apoptosis and inhibit tumor proliferation and metastasis through interactions with miRNAs and proteins. The M1-enhancing property of GAS5 might contribute to its reduced expression in cancer tissues, necessitating further investigation. Overall, research indicates that GAS5 boosts NF-κB-mediated inflammation and shifts macrophage polarization towards M1, while inhibiting GAS5 has the opposite effect in various diseases.

LncRNA-Cox2, located near the COX2 gene [184], is linked with chronic inflammatory diseases and innate immune activation [185,186]. In systemic lupus erythematosus (SLE) patients, serum lncRNA-Cox2 level was elevated more than three-fold and correlated with neurological symptoms [187]. It is highly induced in an NF-κB-dependent manner in activated macrophages and interacts with the switch/sucrose nonfermentable (SWI/SNF) chromatin-remodeling complex [184,188]. This interaction enhances the expression of NF-κB-induced late inflammatory genes [105]. In neuroinflammation, lncRNA-Cox2 promotes NLRP3-inflammasome formation and hinders autophagy by binding and facilitating the p65 subunit’s nuclear transport [104]. However, its role in inflammation is subject to debate, as it appears to suppress inflammation in M1 macrophages and BMDMs in septic mouse models [188,189], indicating a complex influence in inflammatory contexts.

During allergic disease development, a type 2 immune response led by Th2 cells, eosinophils, basophils, and M2 macrophages is predominant [190]. In allergic rhinitis clinical samples and animal models, a downregulation of the lncRNA miR222 host gene (MIR222HG) was observed, linking it to the type 2 response [110]. MIR222HG is upregulated in M1 macrophages but downregulated in M2 macrophages, with its overexpression reducing M2 polarization and allergic inflammation. MIR222HG activates the NF-κB pathway via the miR-146a-5p/TRAF6 axis [110]. This suggests that MIR222HG downregulation in allergic rhinitis contributes to pathogenesis by favoring M2 macrophage polarization, and its overexpression might alleviate the condition.

In contrast to MIR222HG, NF-κB interacting lncRNA (NKILA) directly promotes M2 macrophage polarization by suppressing NF-κB activation in asthma [112]. NKILA has been found to have anti-inflammatory effects and dysregulation of its expression has been observed in cancers as well as immune-related disorders such as epilepsy, osteoarthritis, periodontitis, coronary artery disease, and asthma [112,191]. Overexpression of NKILA alleviated airway hyperresponsiveness and asthmatic mice and reduced macrophage abundance through inhibition of the NF-κB pathway [112]. The inhibitory effect of NKILA on the NF-κB pathway was reported to be through the inhibition of IκB phosphorylation in laryngeal cancer cells.

Celiac disease, characterized by constant NF-κB activation, shows increased cardiac and apoptosis-related lncRNA (CARLR) expression in patient samples [103,192]. CARLR expression rises in THP-1 cells post-LPS stimulation. CARLR interacts with activated NF-κB following IκB dissociation, boosting the expression of cytokines such as IL-1β and COX2, also elevated in celiac disease samples [103]. This suggests a crucial role of CARLR in the disease’s inflammatory response.

NAIL, an evolutionarily conserved lncRNA, exhibits approximately a three-fold increase in expression in samples from patients with inflamed ulcerative colitis compared to those from non-inflamed cases [111]. It activates NF-κB by binding to Wip1 phosphatase, thereby removing Wip1 from its substrates, including p38 MAPK and p65, which play a key role in the inflammatory process of ulcerative colitis.

LncRNA P53-induced transcript (PINT) is upregulated in the intestinal mucosa of ulcerative colitis patients and in inflammatory bowel disease mouse models [107]. In macrophages, LPS induces PINT expression in an NF-κB-dependent manner. Acting as a scaffold, PINT enables the binding of p65 and EZH2, a histone methyltransferase and epigenetic regulator, to NF-κB sites on target promoters [193]. This complex stimulates TNF-α expression while inhibiting other pro-inflammatory mediators like CCL2/7 and intercellular adhesion molecule-1.

MALAT1, with high expression in severe acute pancreatitis patients’ plasma and corresponding mouse models, plays a critical role in this condition [124,194]. Its suppression reduces tissue injury and inflammation. Additionally, MALAT1 is found in serum EVs of acute pancreatitis patients, stimulating macrophages to activate TLR4/NF-κB signaling and M1 polarization via the miR-181a-5p/HMGB1 axis [124,194]. In endothelial progenitor cell-derived exosomes, MALAT1 triggers BMDM differentiation into osteoclasts, binding to miR-124 [195]. High glucose levels in macrophages also prompt MALAT1-containing exosome secretion [196], highlighting its role in cellular communication through EVs.

These studies underscore the pivotal roles of various lncRNAs in autoimmune, hypersensitivity, and inflammatory conditions. These lncRNAs modulate NF-κB activity, either directly or indirectly, impacting macrophage polarization and immune responses. The intricate relationship between these lncRNAs and macrophage NF-κB dynamics opens avenues for targeted therapies in such diseases. Given NF-κB’s association with inflammation, macrophage polarization, apoptosis, and pyroptosis, modulating lncRNA functions could favorably alter disease progression by influencing NF-κB activity.

## 6. Discussion

Research targeting lncRNAs in disease treatment shows promise, particularly through suppression or overexpression in animal models. For instance, suppressing MALAT1 with siRNA mitigates sepsis-related inflammation [43,44,45], while its overexpression exacerbates atherosclerosis severity [91]. Similarly, altering the M1/M2 macrophage polarization balance can influence disease progression. Knockdown of GAS5 in diabetic wound healing models encourages M1-to-M2 transition, enhancing wound healing [177], and its suppression reduces EAE progression by inhibiting M1 polarization [179]. Reducing lncRNA-Cox2 in HCC models strengthens M2 macrophage polarization, promoting tumor growth [197].

LncRNA-targeting therapies primarily use nucleic acid-based methods like antisense oligonucleotides (ASOs), RNA interference with siRNA or shRNA, and innovative approaches like CRISPR/Cas and exosome-mediated transfer [198,199]. While clinical trials have mainly focused on miRNAs, the exploration of lncRNAs as diagnostic markers and therapeutic targets is growing. For example, MALAT1 and lncRNA prostate cancer antigen 3 are being studied as diagnostic markers for prostate cancer. Trials involving ASncmtRNA-targeting ASOs, such as Andes-1537 [200], are assessing safety and efficacy in various cancers, indicating a progressing field in lncRNA-based treatments.

Contrary to its well-documented role in promoting inflammation, emerging evidence suggests that NF-κB may also be involved in anti-inflammatory responses within macrophages, highlighting the complex role of NF-κB in immune regulation [201]. This dual functionality depends on the context, including the specific cell type, the nature of the stimuli, and the timing of NF-κB activation. For example, many negative regulators of TLR signaling such as A20, IκB, and protein–tyrosine phosphatases are direct targets of NF-κB signaling [201]. SREBP1, a transcription factor influenced by NF-κB, contributes to the resolution of pro-inflammatory TLR4 signaling by reprogramming fatty acid metabolism in macrophages. This reprogramming leads to the production of anti-inflammatory fatty acids, further indicating a mechanism through which NF-κB may support anti-inflammatory responses [202]. These anti-inflammatory actions of macrophage NF-κB may complicate future efforts to manipulate its activity for disease treatment, necessitating careful design in therapeutic approaches.

The expression of lncRNAs that regulate macrophage NF-κB activity is implicated in various human diseases. Animal studies targeting these lncRNAs have yielded promising results, positioning them as potential future therapeutic targets. The primary focus will be on reducing macrophage inflammation in chronic inflammatory diseases, hypersensitivity, autoimmune disorders, and cancer. A secondary objective will involve balancing M1 and M2 macrophage polarization in these conditions. While the use of NF-κB-targeting lncRNAs as therapeutic agents is still in its early stages, the critical role of macrophage NF-κB in disease pathogenesis makes these targets particularly promising.

## 7. Conclusions and Future Directions

This review has comprehensively explored the dynamic interplay among lncRNAs, NF-κB activation, and macrophage function across various diseases. It elucidates how lncRNAs and NF-κB are mutually regulatory within a complex network that is pivotal for both genomic and epigenomic regulations. The regulation of NF-κB by lncRNAs, achieved through both direct and indirect methods affecting NF-κB’s expression or activity, is essential for cellular homeostasis and the response to environmental stimuli. However, dysregulation in this system can lead to the development of various diseases. This review underscores the significant roles of specific lncRNAs in modulating inflammatory responses and macrophage polarization, positioning them as potential biomarkers and therapeutic targets. The divergent roles of certain lncRNAs across different disease contexts underscore the complexity of their functions, thereby emphasizing the need for further research to enhance our understanding and facilitate their clinical application.

Future research should focus on unraveling the detailed mechanisms of how lncRNAs influence NF-κB pathways and macrophage polarization in various diseases. The development of more specific and effective lncRNA-targeted therapies, possibly using advanced techniques like CRISPR/Cas9 and exosome-mediated delivery, is another crucial direction. Clinical trials focusing on the therapeutic potential of lncRNAs and their safety and efficacy in human diseases will be vital. Additionally, exploring the role of lncRNAs in other immune cells and their interaction with macrophages could provide a more comprehensive understanding of immune regulation and disease pathogenesis.

## Data Availability

The data used in this article are sourced from materials mentioned in the References section.

## Abbreviations

ABCA1, ATP-binding cassette subfamily A member 1; AML, acute myeloid leukemia; ASncmtRNA, antisense noncoding mitochondrial RNA; ASO, antisense oligonucleotide; BMDM, bone marrow-derived macrophage; BST2, bone marrow stromal cell antigen 2; BTK, Bruton’s tyrosine kinase; CAD, coronary artery disease; Cas, CRISPR-associated protein; COX, cyclooxygenase; CRISPR, clustered regularly interspaced short palindromic repeats; CRNDE, colorectal neoplasia differentially expressed; DDA1, DET1- and DDB1-associated 1; EAE, experimental autoimmune encephalomyelitis; EV, extracellular vesicle; EZH2, enhancer of zeste homolog 2; FGD5-AS1, FGD5 antisense RNA 1; GAS5, growth arrest-specific 5; HCC, hepatocellular carcinoma; HMGB1, high mobility group box 1; HOTAIR, HOX transcript antisense RNA; HOTTIP, HOXA transcript at the distal tip; HUVEC, human umbilical vein endothelial cell; IL, interleukin; LDL, low-density lipoprotein; lncRNA, long noncoding RNA; LPS, lipopolysaccharide; MALAT1, metastasis-associated lung adenocarcinoma transcript 1; MAPK, mitogen-activated protein kinase; MI, myocardial infarction; miRNA, microRNA; MMP, matrix metalloproteinase; MyD88, myeloid differentiation primary response 88; NEAT1, nuclear paraspeckle assembly transcript 1; NF-κB, nuclear factor kappa-light-chain-enhancer of activated B cells; NLRP3, NLR family pyrin domain containing 3; oxLDL, oxidized-LDL; PBMC, peripheral blood mononuclear cell; PINT, P53-induced transcript; PRC2, polycomb repressive complex 2; PVT1, plasmacytoma variant translocation 1; ROS, reactive oxygen species; shRNA, short hairpin RNA; siRNA, small interfering RNA; SMAD3, small mothers against decapentaplegic homolog 3; SNHG, small nucleolar RNA host gene; SOCS, suppressor of cytokine signaling; STAT, signal transducer and activator of transcription; TAK1, TGF-β-activated kinase 1; TAM, tumor-associated macrophage; TLR, Toll-like receptor; TNF-α, tumor necrosis factor-α; TRAF6, TNF receptor-associated factor 6; ULK1, Unc51-like autophagy activating kinase 1; XIST, X-inactive specific transcript.
